# Conservative Medicine

**Published:** 1862-10

**Authors:** Austin Flint

**Affiliations:** Prof. of the Principles and Practice of Medicine in the Bellevue Hospital Medical College, N. Y., and in the Long Island Hospital


### SELECTED.

### CONSERVATIVE MEDICINE.

By AUSTIN FLINT, M. D.,
*Prof. of the Principles and Practice of Medicine in the Bellevue Hospital Medical College, N. Y., and in the Long Island Hospital.*

(Concluded from September Number.)

Clinical experience has rendered it doubtful whether the antiphlogistic treatment exerts much effect on the intensity of inflammation, its results, or its duration. Conservatism, therefore, dictates a careful weighing of the evils of the treatment againt the chances of its usefulness as regards these objects.

It is not settled by experience that this treatment, carried to a greater or less extent, is always in no measure efficacious. Hence, there is room for difference of opinion, and the practice of different physicians will differ. The discriminating practitioner, who, although satisfied of the evils of the indiscriminate employment of antiphlogistic measures, believes in their ability, if judiciously employed, will be guided, in withholding or resorting to them, by the circumstances belonging to individual cases. And here it is that his practical knowledge, judgment, and tact are brought to bear on the management of inflammatory affections. Conservatism will dictate to such a practitioner not to employ blood-letting, etc., when the inflammatory affection, from its seat and degree of intensity, involves no danger, and when there is reason to suppose that it may pass through its course favorably, without active interference. Conservatism will dictate the same policy when all the local results to be expected from the progress of inflammation have already taken place, and the restorative processes only remain —a condition illustrated by the second stage of pneumonia, when all the exudation that is to occur has occurred, and the recovery involves only the absorption of the morbid deposit. Conservatism will dictate the same line of conduct in all cases of disease in which more danger is to be expected from failure of the powers of life, than from lesions incident to the local affection.

The value of therapeutic agencies is, of course, to be determined by experience. Developments in the progress of pathology, however, contribute to our knowledge of therapeutics, not only by giving direction to clinical observation, but by harmonizing with the conclusions drawn from the latter. It is interesting to note the consistency of the practical views now generally held as regards antiphlogistic measures, with late developments respecting the origin of certain inflammations. Inflammations not traumatic were formerly considered, and are now often called, spontaneous. We may use this term conventionally as distinguishing a local disease not referable to any obvious local cause, but, strictly, it is an absurdity to say that any disease is spontaneous. Every local affection must involve an adequate morbific agency acting on the part affected. It is true that our present knowledge does not enable us generally to appreciate the nature, sources, and the *modus agendi* of the proximate causes of inflammatory affections, but we have acquired, of late years, some information important in itself as a basis for analogical reasoning. Clinical observation has shown that the accumulation of urea in blood is apt to lead to inflammation of serous structures. This we know, and it is a rational supposition that urea (or the products of its decomposition) induces inflammation, by acting directly on these structures. There are sufficient grounds for believing that the local inflammations occurring in gout and rheumatism are due to the looal action of a *matereis morbi* in the blood; perhaps the uric acid in the former, and the lactic acid in the latter of these diseases. Reasoning by analogy, we may expect with considerable confidence that future researches will show the so-called spontaneous inflammations generally to be produced in a similar manner. And with this view of their production, we should rationally expect great results, not so much from the antiphlogistic treatment, as from measures addressed to the morbid conditions of the blood which underlie the local manifestations of disease. To ascertain these morbid conditions in different diseases, to prevent the introduction or accumulation of morbific material in the blood, to neutralize the poisonous properties of this material, by causing the promotion of innocuous combinations, prevent the organico-chemical changes which its presence induces, (catalysis,) or to eliminate it through the emanations of the body —these are the great objects of therapeutics at the present day, which harmonize with the late revelations of pathology. Without stopping to inquire how far these objects have been obtained, it is to be remarked that they are obviously conservative, involving, as they do, protection against internal agencies inimical to life and health.

Conservative medicine thus dictates, in inflammatory affections, proper discrimination in the employment of the so-called antiphlogistic measures, which, if failing to exert a controlling influence, are necessarily hurtful, and, it may be, destructive, by impairing the vital forces. It also dictates the judicious use of remedies addressed to the internal causative conditions pertaining to the blood, so far as our present knowledge extends into these most important provinces of pathology and therapeutics. But this is not all. Conservatism often demands that the vital powers shall be sustained. Sustaining measures of treatment, practically considered, consist of tonics, alcoholic stimulants, and nutritious diet. We will not inquire as to the *rationale* of the operation of these measures. Suffice it to say, clinical experience shows abundantly that they lessen the degree to which the vital forces would otherwise be impaired by disease, and may prevent a fatal termination of disease by exhaustion. I have already admitted that the phrase "powers" or "forces of life" is metaphorical. Life is not an entity. But with a fair understanding that this personification of a combination of conditions, as yet but imperfectly understood, is merely for convenience, it is unobjectionable. The powers or forces of life enable the system to bear up under disease, to resist it successfully, and recover from it. On the other hand, we may say that disease destroys by overcoming the powers of life, whenever death takes place by asthenia or exhaustion. Every sagacious practitioner knows that certain symptoms, no matter with what disease they are associated, denote failure of the vital powers, or inability to resist disease. He estimates the amount of danger by these symptoms, among which those referable to the circulation are especially important. He often bases his prognosis far more on these symptoms than on the nature and extent of the local affection. Every practitioner knows that an inflammation, the same in all respects, so far as the local affection is concerned, in different persons, affects the vital forces differently. Take, for example, pneumonia, extending over the same space, and inducing an equal amount of changes, which physical exploration enables us to determine with accuracy: one patient manifests little disturbance of the system, and no symptoms denoting danger, while another patient will succumb to the disease. Every practitioner knows that some persons, who, in health, present no evidence of a lack of vigor, have very little ability to resist severe disease. They are quickly destroyed by affections which other persons readily endure, and endure, perhaps, without much inconvenience. Of course, these facts are explicable, but not with our present knowledge, and, until explained, it answers to refer them to differences as regards the vital powers or forces. They are facts of not a little practical importance.

Conservatism dictates sustaining treatment in any inflammatory affection whenever the symptoms denote failure of the vital powers, whether the period be early or late in the course of the affection. This treatment is to be pursued vigorously in proportion to the rapidity of the failure and the amount already taken place. It is important, in all dangerous affections, to watch for the first evidence of failure, and to lose no time in resorting to supporting measures. Such is the influence of traditional ideas, that these measures are frequently delayed from a timidity which experience is sure to remove. It is far wiser to enter on the use of tonics, stimulants, and a nutritious diet to early, or when not required, than to incur risk of delay, or their omission when required. In the one case, the liability of harm is small; but in the latter, lost time, which cannot be regained, may have been of immense importance to the patient. So far from incurring risk of damage from delay, the wise practitioner will anticipate the indications for support, and forestall the failure which he knows would otherwise occur. Physicians, however devoted to the antiphlogistic treatment of inflammations, have generally recognized the importance of supporting measures to "obviate tendency to death." When the flame of life is reduced to a glimmer, they would prevent it, if possible, from going out. Does not common sense teach that measures which may prove serviceable under these circumstances, would have proved much more so when the danger was less imminent? Is it not better policy to endeavor to keep the lamp of life burning brightly, than to depend on efforts to restore the flame when nearly extinguished? In cases involving danger to life, the importance of sustaining treatment is to be measured by these questions: Is the chief danger failure of the vital powers, and how great is the danger from this source? In cases not involving danger to life, the importance of support has referencc to the duration of the disease, the rapidity of convalescence, and the condition of the recovery. The advantages derived from the proper application of conservatism, as regards sustaining treatment, in all operations, by no means consist exclusively in a reduced state of mortality, but also in a speedy and rapid convalescence, and in the completeness of the restora tion to health.

These remarks have had reference more especially to acute inflammations. Chronic inflammation affecting an important part may continue for a greater or less period, and recovery finally be complete; but during its continuance the powers of life are more or less impaired. It may destroy life by leading to incurable lesions, or by its protracted duration; in either case death usually taking place by slow asthenia. Under all circumstances, the affection is less likely to be prolonged, serious changes of structure are less likely to take place, and a fatal termination is postponed in proportion as the vital powers are preserved. Conservatism, therefore, dictates not measvres to reduce, but those which sustain the powers of life in chronic inflammations. It dictates measures to develop appetite and improve the digestive processes, abundant nutritive supplies, and, in short, the remedies and hygienic means which invigorate and strengthen the body. The "building up" treatment, as it is significantly called, has contributed largely to the more successful management of chronic affections since the days of Broussaisism. Some of the most striking examples of the efficacy of this treatment which I have seen have cases of chronic pleurisy, in which speedy and progressive improvement followed directly the substitution of this treatment for measures opposed to the principles of conservatism. These examples are the more satisfactory becapse, by means of physical signs, the improvement within the chest was accurately determined at the same time that the local and general symptoms denoted a favorable change. Certain cu taneous inflammations, and cases of opthalmia, the parts in these affections being open to inspection, also afford examples not less striking.

Conservatism has been practically more fully applied to the management of essential fevers than of inflammatory affections. Since nearly all pathologists have admitted the essentiality of fever, and since physicians have ceased to agree with Southwood Smith in regarding inflammation as an almost concomitant and the chief source of danger in fever, the importance of preserving and sustaining the powers of life has been more and more appreciated, and, at the present moment, with the most intelligent practitioners, these are the leading objects in the treatment. In this remark I refer especially to fevers having a self-limited career, and not arrested by abortive measures. The periodical fevers are controllable by remedies having a special efficacy. These remedies are conservative, acting in an imperceptible manner, and, given within proper limits, producing no destructive or injurious effects, even if not indicated. It is a curious fact, that the fevers which we are able to arrest with great certainty, *i. e.*, the periodical fevers, continue indefinitely if not arrested, and return, sooner or later, and more or less frequently, in the majority of cases; whereas the fevers which we cannot arrest with any certainty, if at all, *i. e.*, the continued fevers, the eruptive, and yellow fever, have a fixed duration, and, as a rule, are experienced only once. It is not without the bounds of a reasonable expectation that the means of arresting the last named fevers will hereafter be discovered. Reasoning by analogy, and from the pathological views now generally entertained, the means for this end must act by neutralizing a morbid material in the blood, or effecting its elimination; acting, therefore, in accordance with the principle of conservatism.

Tonic remedies, alcoholic stimulants, and nutritious diet are the measures for maintaining the vital forces during the course of the essential fevers. The importance of these measures is now so generally admitted as hardly to require argument or advocacy. The only questions for discussion relate to circumstances indicating their employment, the extent to which they are to be carried, and various details connected with their use. The discussion of these questions does not fall within the scope of this article. I may be indulged, however, in a few remarks on some interesting points connected with the subject.

One of these is the wonderful tolerance of alcoholic stimulants in certain cases of fever. Examples have been of late so often repeated, and are so generally familiar, that they need not be cited. How much at variance are the effects of pints, or even quarts, of spirit, given daily, with those produced in health! And how fully does fact, as well as analogous facts relating to the action of opium and other remedies, illustrate the liability to error in judging of the operation of therapeutic measures in disease from experimental observations in healthy persons! How surprised, but a few years ago, would have been the therapeutist if told that the action of alcohol, under certain morbid conditions, is in fact sedative; in other words, that, in certain cases of typhus or typhoid fever, two or three ounces of spirit given hourly lessen the frequency of the pulse, diminish the heat of skin, and render the mind more clear! But the past history of medicine shows a tendency to push prevailing ideas to an extreme, against which the prudent physician should endeavor to guard himself. There is danger now of carrying the use of alcohol to an injudicious and dangerous extent. The principle of conservatism should be the guide. The object is to sustain the vital forces. The toleration is in proportion to the need of this sustaining agent. If it be used excessively in all cases, without discrimination, it will sometimes do harm, and life may be destroyed by alcoholic poisoning. We have already seen that, within certain limits, alcohol is eminently a conservative remedy, because even when not indicated, it is not destructive, and its operation is transient; but beyond certain limits its effects may be poisonous, provided it does not fulfill indications showing that the system is tolerant of quantities which would be dangerous in health. Let the indications, then, in individual cases, be carefully observed, and let the effects be carefully noticed, so as not to violate, but conform, to the rule of conservatism.

Some interesting points are connected with the dietetic management in cases of fever. In perfect health, the wants of the system for alimentary supplies are expressed by hunger and appetite. Common observation, however, teaches us that these sensations are not essential as prerequisites to digestion and nutrition. Almost every one has experienced a state, certainly abnormal, but not dependent upon any well-defined disease, and not interfering with the usual habits of mental and physical activity, in which food is taken habitually for a greater or less period without hunger or appetite, and nevertheless properly assimilated. Intense mental preoccupation and persisting depressing emotions may involve such a state. During the career of fevers, usually, hunger and appetite are wanting, but it is not to be inferred therefrom that the ability to appropriate nutriment is lost. Some have reasoned that the absence of the desire for food is always evidence of its not being needed, and a comparison has been made between the morbid condition, in this regard, in the essential fevers, and the natural state of hybernation. But the analogy holds good only as respects the disinclination for food. In hybernation, the respirations, the heart's action, muscular movements, and the functional exercise of all the organs, are reduced to the lowest point compatible with the preservation of life. In fever, the respirations are far oftener increased than diminished in frequency, and more oxygen enters the system than in health; the heart beats with unwonted frequency, muscular action is not wanting, and in the more frequent respiratory movements it is above the healthy standard; the mental faculties are sometimes morbidly active, and, from the absence of sleep, often more continuously so than in health; calorification is increased, and various functions of the body manifest disordered activity. It seems sufficiently clear that no practical inferences are to be drawn from a comparison between the arrest of hunger and appetite in fever, and the suspension of these sensations in hybernation. In hybernation the system has no need for alimentary supplies, and, hence, there is no physiological expression of the want of them. In fever, the morbid conditions prevent the feeling of this want, although the need of alimentary supplies continues.

The correctness of the statement just made rests on clinical observation. Patients with fever, taking food without inclination, and even with repugnance, retain it, and no disturbance is produced by its ingestion; the fæcal evacuations may present a normal appearance, and, in some cases, in which a nutritious diet has been entered upon after the disease has existed for some time, there is an evident increase of muscular strength, although the career of the fever continues. These are clinical facts. And the conclusion is, digestion and nutrition are not incompatible with the state of fever, although hunger and appetite may be wanting. The faculty of perceiving these sensations is impaired or lost in consequence of the morbid condition of the nervous system, and, hence, they cease for the time to express the demands of the system. The perceptions are often so blunted that the mind takes no cognizance of other wants of the system. The urine is allowed to accumulate in the bladder, and, with the tongue desiccated, the patient manifests no desire for drink. Fatigue from lying continuously in the same position is not complained of. Local complications of the disease are not accompanied by pain. Under these circumstances, it is cousistent that the sensations of hunger and appetite should not be experienced. The perceptive faculties, however, sometimes are not so much impaired as they appear to be. Desires and feelings may not be manifested from an extreme reluctance to make any exertion. Thus, patients not unfrequently drink with avidity when the cup is brought to their lips, who make no complaint of thirst; and in some cases, food, when presented, is also taken with relish.

In sustaining the powers of life in fevers, then, (and also in certain other diseases,) the physician is not to be restrained by the absence of hunger and appetite. He is to act with reference to the wants of the system by endeavoring to secure the ingestion of food, concentrated, containing the necessary variety of alimentary principles, and ample in quantity. Here, too, as with regard to alcoholic stimulants, it is far better to begin earlier than is needed, than run any risk of delay, and to give more aliment than is required than not enough. An appreciation of the importance of alimentation in fevers is among the most important of the recent improvements in practice which exemplify the spirit of conservatism. But in all acute diseases, whenever the chief end of treatment is to support the powers of life, a nutritious diet is essentially important, and the same rules with regard to dietetic management are alike applicable.

It will suffice to notice the application of conservatism to those chronic affections collectively which destroy by gradual inroads upon the powers of life. In this class are grouped such affections as carcinoma, tuberculosis, chronic dysentery, cirrhosis, and Bright's disease. It is sufficiently clear that, with a view to the prolongation of life, when recovery is not expected, the great object is to retard, as much as possible, the failure of the vital forces. If we cannot "build up," we may do much to delay the progress of destruction. Evident as this is, it is not sufficiently appreciated by all practitioners.

Patients affected with incurable diseases are too often abandoned to merely palliative remedies, the fatal issue being considered as merely a question of time, and, therefore, not of much importance. This question of time, however, may be highly important to patients and their friends. To aid in the cure of diseases is, undoubtedly, the first aim of the physician; and next to this, when a cure is not to be effected, comes the prolongation of life, with health more or less impaired. The last of the grand objects of practice are palliation and euthanasia.

In the management of any incurable affection, conservatism dictates the measures which, in general terms, contribute to keep the body in the best possible condition compatible with the continuance or progress of the disease. In this way not only the inroads of the disease on the powers of life, but the destructive lesions in the parts affected, are often stayed. It may be assumed to be a rule in pathology, that a local affection involving structural changes is less likely to progress with rapidity, the closer the approximation to health in all other respects. The practice which conservatism dictates in such cases is in accordance with this rule. An incurable lesion is sometimes so completely held in abeyance, and the system is rendered so tolerant of its continuance, that life may be preserved indefinitely, although a vital organ be affected. We meet with cases in which depositions of tubercle and carcinoma remain for a long time non-progressive and nearly innocuous. The conservative practice, moreover, favors those retrogressive changes by which even the disease just named may eventuate a cure.

To consider the measures for keeping the body in the best possible condition, would be to enter on a large but immensely important domain of practical medicine. I must content myself with saying that they consist, first, of a nutritious diet; next, of remedies to strengthen and invigorate; and last, of hygienic influences directed to the same end. The hygienic influences comprise exercise and everything relating to regimen, change of climate, mental diversion and encouragement —in short, whatever can be brought to bear favorably upon the welfare and vigor of the system. The hygienic is certainly not inferior in importance to the medical treatment, and here it is that the judgment and tact of the successful practitioner are especially brought into requisition.

A comparison of cases of pulmonary tuberculosis now and twenty-five years ago, illustrates the importance of the practical views just presented. The management of this disease twenty-five years ago was certainly not in accordance with the principle of conservatism. The measures employed, medicinal and hygienic, were, indeed, directly opposed to this principle. The antiphlogistic system of treatment was often adopted, under the belief that inflammation was the most impotent element of the local affection. Blood-letting, cathartics, mercurialization, severe counter-irritation, were considered as remedial, and to these were conjoined low diet and confinement within doors. Now, pulmonary tuberculosis is not cured in the majority of cases, although it is not incurable; and there is reason to believe that the proportion of cures is considerably larger than under the treatment just referred to. But, directing attention to the incurable cases, under the plan of treatment generally pursued at the present time, which is eminently conservative, how striking the contrast! Formerly, the instances of rapid progress of the disease were more numerous, and it almost invariably advanced with a steady march, rarely occupying many months in completing its fatal career. Patients were usually confined to the bed for weeks before death, lingering on the borders of the grave, suffering from extreme debility, bed-sores, aphthæ, and colliquative diarrhœa. It was difficult to conceive of a picture more distressing and repulsive than that of an unfortunate being in the last stage of consumption. Conservatism has done much towards ameliorating the condition of consumptives, even when it is hopeless as regards recovery. Cases of so-called galloping consumption are less frequent. Life is not unfrequently prolonged and made comparatively comfortable for years. It is not uncommon to meet with instances of a considerable deposit of tubercle remaining quiescent or progressing very slowly, and the patient able to engage in the active occupations and enjoyments of life. Even when the disease is progressing to a fatal termination, the strength is usually so far preserved that a bedridden consumptive is now rarely seen, and it is not uncommon for patients to be out of doors almost up to the hour of their death. I appeal to those whose medical experience has extended over a quarter of a century for the truthfulness of this comparison.

In concluding these fragmentary remarks, let it be borne in mind that, important as is conservatism in medical practice, it is by no means inconsistent with the employment of efficient therapeutic agencies in the management of diseases. The conservative surgeon does not hesitate to use the knife and dismember the body, when convinced that thereby he may save life. So the conservative physician resorts without hesitation to his potential remedies—not less potent for good or evil than the scalpel—whenever he sees clearly that they will contribute to the safety and welfare of the patient.—*N. A. Med. Chir. Rev.—Am. Med. Monthly.*

